# Water and sodium intake habits and status of ultra-endurance runners during a multi-stage ultra-marathon conducted in a hot ambient environment: an observational field based study

**DOI:** 10.1186/1475-2891-12-13

**Published:** 2013-01-15

**Authors:** Ricardo JS Costa, Ana Teixeira, Luis Rama, Abigail JM Swancott, Lisa D Hardy, Benjamin Lee, Vera Camões-Costa, Samantha Gill, Jessica P Waterman, Emily C Freeth, Edel Barrett, Joanne Hankey, Slawomir Marczak, Encarna Valero-Burgos, Volker Scheer, Andrew Murray, Charles D Thake

**Affiliations:** 1 Department of Health Professions, Coventry University, Priory Street, Coventry, CV1 5FB, United Kingdom; 2 Sport & Exercise Science Applied Research Group, Coventry University, Priory Street, Coventry, CV1 5FB, United Kingdom; 3 Faculty of Sport Science & Physical Education, Coimbra University, Pavilion 3, Santa Clara, 3040-156, Portugal; 4 School of Psychology, Bangor University, Penralt Road, Bangor, LL57 2AS, United Kingdom; 5 Emergency Department, Vinalopo Salud Hospital, Calle Tonico Sansano Mora 14, Elche, 03293, Spain; 6 Sports Medicine Department, University of Heidelberg, Im Neunheimer Feld 710, Heidelberg, 69120, Germany; 7 SportsScotland Institute of Sport, Airthrey Road, Stirling, FK9 5PH, United Kingdom

**Keywords:** Water, Drinking, Beverages, Total body water, Dehydration, Euhydration, Hyponatraemia, Carbohydrate

## Abstract

**Background:**

Anecdotal evidence suggests ultra-runners may not be consuming sufficient water through foods and fluids to maintenance euhydration, and present sub-optimal sodium intakes, throughout multi-stage ultra-marathon (MSUM) competitions in the heat. Subsequently, the aims were primarily to assess water and sodium intake habits of recreational ultra-runners during a five stage 225 km semi self-sufficient MSUM conducted in a hot ambient environment (T_max_ range: 32°C to 40°C); simultaneously to monitor serum sodium concentration, and hydration status using multiple hydration assessment techniques.

**Methods:**

Total daily, pre-stage, during running, and post-stage water and sodium ingestion of ultra-endurance runners (UER, *n* = 74) and control (CON, *n* = 12) through foods and fluids were recorded on Stages 1 to 4 by trained dietetic researchers using dietary recall interview technique, and analysed through dietary analysis software. Body mass (BM), hydration status, and serum sodium concentration were determined pre- and post-Stages 1 to 5.

**Results:**

Water (overall mean (SD): total daily 7.7 (1.5) L/day, during running 732 (183) ml/h) and sodium (total daily 3.9 (1.3) g/day, during running 270 (151) mg/L) ingestion did not differ between stages in UER (*p* < 0.001 *vs*. CON). Exercise-induced BM loss was 2.4 (1.2)% (*p* < 0.001). Pre- to post-stage BM gains were observed in 26% of UER along competition. Pre- and post-stage plasma osmolality remained within normal clinical reference range (280 to 303 mOsmol/kg) in the majority of UER (*p* > 0.05 *vs*. CON pre-stage). Asymptomatic hyponatraemia (<135 mmol/L) was evident pre- and post-stage in *n* = 8 UER, corresponding to 42% of sampled participants. Pre- and post-stage urine colour, urine osmolality and urine/plasma osmolality ratio increased (*p* < 0.001) as competition progressed in UER, with no change in CON. Plasma volume and extra-cellular water increased (*p* < 0.001) 22.8% and 9.2%, respectively, from pre-Stage 1 to 5 in UER, with no change in CON.

**Conclusion:**

Water intake habits of ultra-runners during MSUM conducted in hot ambient conditions appear to be sufficient to maintain baseline euhydration levels. However, fluid over-consumption behaviours were evident along competition, irrespective of running speed and gender. Normonatraemia was observed in the majority of ultra-runners throughout MSUM, despite sodium ingestion under benchmark recommendations.

## Introduction

Multi-stage ultra-marathon (MSUM) events, commonly conducted in hot ambient conditions (≥30°C) and routed over undulating desert based terrains, expose ultra-runners to exercise-heat stresses with minimal external support. Due to the well established association between compromised hydration status and decrement in endurance exercise performance [1], programming of individualised hydration strategies before and during MSUM aimed at maintaining euhydration during exercise-heat stress is of prime importance [2]. Maintaining euhydration during consecutive days of endurance running in hot ambient conditions is reported to attenuate cardiovascular and thermoregulatory strain, assists heat acclimatisation, and mitigates clinically significant conditions (e.g. exertional heat illnesses) from occurring [3-5].

During MSUM, besides the exposure to exercise-heat stress on consecutive days (normally ranging from 5 to 8 days), ultra-runners need to consume sufficient water (through foods and fluids) to maintain euhydration throughout competition. In some cases (e.g. self-sufficient MSUM) ultra-runners must ration water supplies (e.g. ~12 L/day provisions), enforcing a potential barrier to maintaining euhydration. Indeed, anecdotal evidence suggests ultra-runners may not be consuming sufficient water through foods and fluids to support consistent maintenance of euhydration along MSUM competition (2009 Al Andalus Ultimate Trail, Loja, Spain). This may possibly due to the lack of nutritional education, ultra-endurance sports cultural trends, time limitations and motivation for appropriate food-fluid preparation and consumption [6,7]. Additionally, practical real-life factors (e.g. dysfunction to thirst and palate sensations, suppressed appetite, competition anxiety, gastrointestinal distress, and/or fluid disinterest) may also limit total water ingestion during consecutive days of exercise-heat stress [6-12]. However, a comprehensive assessment of water ingestion and hydration status of ultra-runners during MSUM conducted in a hot ambient environment has not previously been conducted to confirm these possibilities.

Fluid intake recommendations for endurance exercise have previously been proposed [1,13-15]. For consecutive days of endurance exercise in the heat, it is recommended to initiate exercise in a euhydrated state, and consume sufficient fluids (potentially using thirst as an indicator for fluid consumption) during exercise to prevent substantial depletions in body water [1,13-15]. BM losses ≥2% have previously been linked to changes in haemorheology, metabolic dysregulation, heat intolerance, cardiovascular strain, and subsequent inability to maintain exercise workload [1,5,13]. Interestingly, BM losses ≥2% are a common feature amongst highly trained endurance runners during competition [16,17]. It is suggesting that BM loss, to a certain extent, during running does not substantial impact on the maintenance of exercise workload in faster runners [16,17].

Adequate dietary sodium and/or the inclusion of sodium in fluids (700 to 1200 mg/L) during exercise and recovery has previously been recommended to stimulate thirst, increase voluntary fluid intake, enhance glucose and water intestinal absorption, optimise extracellular and intracellular fluid balance, and potentially mitigate clinical significant episodes of hyponatraemia from occurring [18-21]. The inclusion of sodium within ingested fluids and/or sodium supplementation during exercise is a common practice amongst endurance athletes, and a persistent belief of essential requirement amongst the ultra-endurance population. It has however recently been proposed that sodium supplementation in fluids is not required during endurance exercise in the heat, since sodium losses (e.g. sweat, urine, and faeces) appear to be attenuated during periods of sodium restriction or deprivation [14,22,23]. However, a comprehensive assessment of sodium ingestion and status of ultra-runners during MSUM conducted in a hot ambient environment has not previously been conducted to confirm appropriate sodium ingestion advice to this population.

Due to the exceptional fear of dehydration commonly surrounding the ultra-endurance population, current hydration advice given to ultra-runners at the competition location is largely focused on consuming ‘*as much fluid as tolerated*’ (plus inclusion of sodium supplementation), with further advice advocating increased drinking frequency to produce a consistent clear urine output along competition. This behaviour potentially poses a threat in promoting clinical manifestations of hyponatraemia in high risk groups, especially runners that may suffer from the syndrome of inappropriate anti-diuretic hormone secretion [24-26]. Moreover, it has previously been acknowledged that runners who complete the designated course using a mixture of walking and running (mean speed ≤8 km/h) demonstrated more fluid over-consumption behaviours than those who predominantly run the entire designated course (mean speed ≥8 km/h) [17,24,26], suggesting higher hyponatraemic risk in slower runners. Interestingly, MSUM is a type of event that nurtures the development of such drinking behaviours in thermoneutral conditions [27], but to date, no study has determined hyponatraemia inducing drinking behaviours or incidence along MSUM competition conducted in hot ambient conditions.

Various methods of determining hydration status have previously been proposed and reviewed, with limitations and strengths being observed in all methods [28,29]. To avoid misinterpretation of hydration status, the use of multiple techniques (e.g. pre- to post-exercise BM change, plasma (P_Osmol_) and urine (U_Osmol_) osmolality, multi-frequency bioelectrical impedance analysis) when assessing hydration status of athletic populations is suggested [28,29]. With this in mind, the aims of the current observational field study were primarily to assess water and sodium intake habits of recreational ultra-runners during a semi self-sufficient MSUM conducted in a hot ambient environment; simultaneously monitor serum sodium concentration and hydration status using multiple hydration assessment techniques, along competition. It was hypothesised that firstly, water intake habits of ultra-runners will not be sufficient to consistently maintain baseline euhydration levels throughout competition; secondly sodium intake will be sufficient to maintain normonatraemia along the MSUM.

## Methods

### Setting

The study was conducted during the 2010 and 2011 Al Andalus Ultimate Trail (http://www.alandalus-ut.com), held during the second week of July, in the region of Loja, Spain. The MSUM was conducted over five stages (five days) totalling a distance of 225 km (Table 1), which was performed on a variety of terrains; predominantly off-road trails and paths, but also included steep and narrow mountain passes, and occasional road. Average running intensity for the MSUM was 7.2 (0.9) METs (SenseWear 7.0, BodyMedia Inc., Pittsburgh, PA, USA). Sleeping arrangements from Stages 1 to 5 included a combination of outdoor tent and village sports hall accommodation.

**Table 1:** Stage time and average speed of UER along MSUM competition

	**UER**	**SR**	**FR**
**Stage description**	**Running time (h:min)**	**Running time (h:min)**	**Running time (h:min)**
	**and speed (km/h)**	**and speed (km/h)**	**and speed (km/h)**
Stage 1: 37 km	4:54 (0:51)	5:29 (0:35)	4:10 (0:28)
503 to 1443 m ASL	7.6 (1.4)	6.8 (0.7)	8.9 (1.0)
T_max_: 32°C; RH_max_: 32%			
Stage 2: 45 km	6:37 (1:20)	7:32 (1:06)	5:38 (0:46)
830 to 1338 m ASL	6.8 (1.3)	6.0 (0.8)	8.0 (1.0)
T_max_: 34°C; RH_max_: 33%			
Stage 3: 40 km	4:59 (0:53)	5:37 (0:39)	4:15 (0:27)
689 to 1302 m ASL	8.0 (1.6)	7.1 (0.9)	9.4 (1.2)
T_max_: 38°C; RH_max_: 37%			
Stage 4: 65 km	7:51 (1:25)	8:52 (1:01)	6:48 (0:54)
671 to 1152 m ASL	8.3 (1.8)	7.3 (1.0)	9.6 (1.7)
T_max_: 40°C; RH_max_: 33%			
Stage 5: 38 km	4:16 (1:05)	4:49 (1:05)	3:35 (0:35)
473 to 1065 m ASL	8.9 (2.1)	7.9 (1.6)	10.6 (1.7)
T_max_: 40°C; RH_max_: 40%			
Total: 225 km	28:03 (4:34)	31:52 (2:51)	24:22 (2:20)
	8.0 (1.4)	7.1 (0.6)	9.2 (0.9)

Mean (SD): UER (*n* = 74); slow runners (SR: MSUM mean speed <8 km/h, *n* = 41); fast runners (FR: MSUM mean speed ≥8 km/h, *n* = 33).

### Participants

After ethical approval from the Coventry University Ethics Committee that conforms with the 2008 Helsinki declaration for human research ethics, a convenience sampling observational cohort was studied, whereby 74 out of 134 ultra-runners entered into the MSUM competition, from various continents around the world (e.g. Europe, Africa, Asia, Australasia, North and South Americas), volunteered to participate in the study [mean: UER (Males *n* = 46, Females *n* = 28): age 41 (8) years, height 169 (14) cm, BM 70 (11) kg, body fat mass 17 (5)%]. Additionally, 12 age and anthropometrically matched individuals who accompanied the UER along the MSUM course as support crews, but did not compete (absence of exercise stress), volunteered to participate in the study as part of the control group [CON (Males *n* = 5, Females *n* = 7): age 35 (13) years, height 167 (9) cm, BM 70 (16) kg, body fat mass 21 (6)%], for comparative purposes only. Additionally, for the purpose of data analysis, participants were divided into two groups. A slow group (SR; final ranking range: 46 to 108), who completed the entire distance of the MSUM using a mixture of walking and running (overall mean speed <8 km/h); and a fast group (FR; final ranking range: 2 to 44), who completed the majority of the MSUM distance running (overall mean speed ≥8 km/h). This criterion was predetermined, and participants were grouped according to their overall race time, prior to data analysis. All participants arrived at the MSUM location ≤48 h prior to the start of Stage 1. Only 28% of participants resided in countries with hot ambient conditions similar to those of the race location (≥30°C) at the time of competition; whilst the remaining 72% of participants resided in countries that presented cold or thermoneutral environmental conditions (≤20°C).

### Study design and data collection

Following participant recruitment and informed consent, preliminary measures were taken to determine participant characteristics. Height was measured by a wall-mounted stadiometer. Baseline BM was determined using calibrated electronic scales (BF510, Omron Healthcare, Ukyo-ku, Kyoto, Japan) placed on a hard levelled surface. Waist and hip circumferences were measured using a standard clinical tape measure by trained researchers. BM and circumference measures were used in conducting multi-frequency bioelectrical impedance analysis (Quadscan 4000, Bodystat, Douglas, Isle of Man, UK), which was used to determine body composition.

The current MSUM was semi self-sufficient, whereby participants (including CON) planned and provided their own foods and fluids (except plain water) along the five days of competition. Participants’ equipment and sustenance was transported to each stage section by the race organisation. Only plain water was provided by the race organisers *ad libitum* during the rest phase throughout competition. Additionally, aid stations along the running phase of competition were situated approximately 10 km apart, and only provided plain water, fruit (oranges and watermelon), and electrolyte supplementation (Elete electrolyte add-in, Mineral Resources International, South Ogden, Utah, US). Participants were advised to adhere to their programmed habitual dietary practices throughout the entire MSUM competition.

Each day, for five consecutive days, running stages commenced at either 08:00 or 09:00 h. All participants consumed their breakfast 2 to 3 h before the start of each stage. Within the hour before the start of each running stage, pre-stage measurements were determined and samples collected. Participants were required to provide a mid-flow urine sample (2^nd^ urine of the day upon waking) into 30 ml universal tubes (HR 120-EC, A & D instruments, Tokyo, Japan), prior to BM measurements. Participants were then required to sit for 10 minutes before whole blood sampling, which was collected by venepuncture without venostasis from an antecubital vein using a 21 G butterfly syringe into one lithium heparin vacutainer tube (6 ml, 1.5 IU/ml heparin; Becton Dickinson, Oxford, UK), one K_3_EDTA vacutainer tube (6 ml, 1.6 mg/ml of ethylenediaminetetraacetic acid; Becton Dickinson, Oxford, UK), and one plain vacutainer tube (4 ml, Becton Dickinson, Oxford, UK). Blood samples were immediately (or after clotting in plain vacutainer samples) centrifuged at 1500 *g* for 10 minutes at 4°C (Heraeus Labofuge 400R, Kendro Laboratory, Langenselbold, Germany); plasma and serum were aliquoted into eppendorfs and stored frozen initially at −20°C during the MSUM competition, prior to transferring to −80°C storage after completion of the experimental procedure. Participants were then required to lay supine for 10 minutes before conducting multi-frequency bioelectrical impedance analysis. During this time, the conduction body points (right hand and foot) were cleaned and air-dried prior to electrode attachment and analysis. BM was re-measured in those participants who needed to urinate before the stage start. Immediately post-stage and before any foods or fluids were ingested, BM was measured, followed by blood sampling, as previously described. Participants were then asked to provide a mid-flow urine sample at their earliest convenience. For consistency, the order and methods of pre- and post-stage measurements and sampling were identical and standardised for all stages.

At the end of each competition day (20:00 to 22:00 h) on Stages 1 to 4, trained dietetic researchers (*n* = 8) conducted a standardised structured interview (dietary recall interview technique) on participants (UER and CON) to ascertain total daily food and fluid ingestion. Due to practical and participant factors, it was not feasible to conduct the daily dietary assessment on Stage 5, since MSUM completion occurred within the duration of Stage 5, not completing a 24 h period. To avoid inter-observer variations, each trained researcher conducted the interview on the same UER throughout the entire MSUM. Participants were educated and instructed to recall in detail all foods and fluids ingested along the competition day, which included specified food and beverage quantities (e.g. g, ml, litres, portions) and qualities (e.g. type of foods-beverages, brands of foods-beverages) ingested for breakfast (pre-stage), during the stage (during running), within the hour after stage completion (post-stage), and from the hour post-stage until sleep. Any foods and fluids that were part of a meal, snack, or beverage that were not ingested (food wastage) were recorded and not included within the dietary analysis procedure. Relevant nutritional information from all food-beverage packages was recorded. The addition of carbohydrate, protein, sodium (including salt), and/or mixed macronutrient supplementations to foods and fluids were also recorded, and used in the dietary analysis.

### Dietary analysis

Total daily water and sodium intakes, water and sodium intakes pre-stage, during running, and post-stage through foods and fluids were calculated through Dietplan6 dietary analysis software (v6.60, Forestfield Software, Horsham, West Sussex, UK) by a trained dietetic researcher. To improve the validity of the dietary analysis, all the nutritional information gathered from food-beverage packages during the interview procedure was entered into the dietary analysis software program. In addition, to improve the reliability of the dietary analysis, all the completed dietary interview logs were blindly analysed by a 2^nd^ trained dietetic researcher. The overall mean inter-observer coefficiency of variation for fluid and sodium variables analysed was 0.7% and 5.2%, respectively.

### Assessment of hydration status

Exercise-induced BM change (pre- to post-stage BM difference) was determined from pre- and post-stage BM values. U_Osmol_ was determined from 300 μl of pre- and post-stage urine samples (Osmocheck, Vitech Scientific, Partridge Green, West Sussex, UK), while the remainder of the urine sample was used to determine urine colour (U_Colour_) as previously reported [30]. Pre- and post-stage P_Osmol_ was determined from 50 μl lithium heparin plasma samples in duplicate by freezepoint osmometry (Osmomat 030, Gonotec, Germany), as previously recommended [31]. Additionally, U_Osmol_/P_Osmol_ ratio was calculated to identify discrepancies between U_Osmol_ and P_Osmol_ along the experimental design [32]. Plasma volume (P_V_) changes were estimated from the haemoglobin and haematocrit content of K_3_EDTA whole blood samples (Coulter Ac T diff, Beckman Coulter Inc., Indianapolis, USA) [33,34], as previously reported [16,35-37]. Pre- and post-stage serum sodium (S_Na_) concentration was determined by ion selective electrode analysis (SpotLyte, Menarini Pharmaceuticals, Florance, Italy). Multi-frequency bioelectrical impedance analysis was used to determine pre-stage total body, extra-cellular, and intra-cellular water. In CON, hydration was assessed pre-Stages 1, 3 and 5 only.

### Data analysis

Data in the text (overall mean value otherwise specified), tables, and figures are presented as mean value and standard deviation (SD). Participants presenting incomplete data sets were removed prior to data analysis (*n* presented in tables and figures for each variable). Fluid intake variables were analysed and are reported as water ingestion through foods and fluids throughout the results section. Considering the potential influence of individual BM differences (especially in relation to gender and training status) on fluid intake variables, data analysis was performed on total values and corrected for BM, as previously reported [38]. Whereas, data analysis for sodium variables were performed for total values and concentration per fluid volume ingested. Descriptive statistics were used to explore quality of fluids ingested. A one-way ANOVA was applied to determine differences in variables between stages, while a two-way ANOVA was applied to determine differences between groups (UER *vs*. CON; SR *vs*. FR; male *vs*. female ultra-runners), and between pre- and post-stage values within stages (SPSS v.17.0.2, Illinois, US). Assumptions of homogeneity were checked, with adjustments to the degrees of freedom and verification by non-parametric equivalents (Kruskal-Wallis and Friedman two-way, respectively) where appropriate. Significant main effects were analysed using post hoc Tukey’s HSD test. The acceptance level of significance was set at *p* < 0.05.

## Results

### Fluid intake

Total daily water ingestion was higher on Stage 4 compared with Stage 3 only in UER (*p* = 0.007) and SR (*p* = 0.034); but did not vary between stages in CON and FR (Table 2). Similarly, when corrected for BM, total daily water ingestion was higher on Stage 4 compared with Stage 3 in UER (*p* = 0.034), and Stages 2 and 3 in SR (*p* = 0.05); but did not vary between stages in CON and FR. Daily water ingestion (total and corrected for BM) was consistently higher in UER compared with CON throughout the MSUM (*p* < 0.001). While, total daily water ingestion was higher (*p* = 0.002) in FR compared with SR on Stage 2 only; but when corrected for BM, no differences were observed. Plain water accounted for 73% (SR 74%, FR 72%) and 41% of total daily fluids consumed by UER and CON, respectively; with remaining fluid ingestion coming from nutrient rich sources, which included (in order of highest consumed volume in UER): soft drinks, fruit juices, carbohydrate solutions, carbohydrate-protein solutions, milks, and protein solutions.

**Table 2:** Total daily water ingestion of UER along MSUM competition

	**Stage 1**	**Stage 2**	**Stage 3**	**Stage 4**	**Overall mean**
Total daily water intake (ml/day)					
UER	7560 (1540)^bb^	7488 (2041)^bb^	7075 (1842)^‡‡bb^	8313 (2282)^bb^	7709 (1499)^bb^
CON	2806 (290)	3411 (201)	3277 (601)	3710 (670)	3301 (163)
SR	7575 (1608)	6986 (2221)^c^	6729 (2025)^‡^	8193 (2581)	7371 (1648)
FR	8520 (1272)	8218 (1510)	7578 (1436)	8487 (1806)	8201 (1110)
Total daily water intake corrected for BM					
(ml/kgBM/day)					
UER	116 (24)^bb^	111 (33)^bb^	106 (31)^‡bb^	124 (39)^bb^	114 (26)^bb^
CON	50 (18)	60 (14)	53 (7)	59 (14)	56 (6)
SR	113 (27)	106 (34)^‡‡^	103 (35)^‡‡^	125 (43)	112 (29)
FR	119 (18)	119 (29)	111 (25)	123 (36)	118 (22)

Mean (SD): UER (*n =* 54); control (CON, *n =* 12); slow runners (SR; MSUM mean speed <8 km/h, *n =* 32); fast runners (FR; MSUM mean speed ≥8 km/h, *n =* 22). ‡‡ *p <* 0.01 and ‡ *p <* 0.05 *vs.* Stage 4; ^bb^*p <* 0.01 *vs.* CON; ^c^*p <* 0.05 *vs.* SR.

Pre-stage water ingestion (total and corrected for BM) was lower on Stages 3 and 4 for UER, and stages 2 and 3 for SR, compared with Stage 1 (*p* < 0.05); but did not vary between stages in FR (Table 3). Pre-stage water ingestion (total and corrected for BM) did not differ between SR and FR within stages. Plain water accounted for 75% (SR 78%, FR 71%) of total fluids consumed pre-stage by UER, with remaining fluid ingestion coming from nutrient rich sources, which included (in order of highest consumed volume in UER): fruit juices, milks, carbohydrate solutions, and carbohydrate-protein solutions.

**Table 3:** Total pre-stage and immediately post-stage total water ingestion of UER along MSUM competition

	**Stage 1**	**Stage 2**	**Stage 3**	**Stage 4**	**Overall mean**
Pre-stage (ml)					
UER	1052 (640)	831 (511)	767 (436)^†^	767 (398)^†^	854 (385)
SR	1112 (967)	782 (521)^†^	784 (432)^†^	803 (401)	870 (387)
FR	967 (530)	902 (500)	742 (452)	715 (398)	831 (390)
Pre-stage (ml/kgBM)					
UER	15.2 (9.1)	12.2 (7.3)	11.4 (7.3)^†^	11.3 (5.8)^†^	12.5 (5.3)
SR	16.2 (9.8)	11.5 (6.7)^†^	11.7 (5.7)^†^	12.0 (5.4)	12.8 (5.0)
FR	13.7 (7.8)	13.1 (8.1)	11.1 (7.6)	10.3 (6.4)	12.0 (6.3)
Post-stage (ml)					
UER	1396 (716)	1171 (667)	1179 (621)	1116 (607)	1214 (504)
SR	1201 (696)	1053 (536)	1014 (464)	1004 (594)	1068 (385)
FR	1604 (667)^c^	1342 (804)	1418 (744)^c^	1280 (602)	1426 (587)^cc^
Post-stage (ml/kgBM)					
UER	20.8 (11.2)	17.7 (10.3)	17.7 (9.4)	16.9 (9.9)	18.3 (7.9)
SR	18.7 (11.8)	16.4 (8.9)	15.8 (8.1)	15.8 (10.4)	16.6 (7.1)
FR	24.0 (9.6)	19.6 (12.0)	20.5 (10.7)	18.6 (9.0)	20.7 (8.6)

Mean (SD): UER (*n =* 54); slow runners (SR; MSUM mean speed <8 km/h, *n =* 32); fast runners (FR; MSUM mean speed ≥8 km/h, *n =* 22). † *p <* 0.05 *vs.* Stage 1; ^cc^*p <* 0.01 and ^c^*p <* 0.05 *vs.* SR.

**Figure 1:**
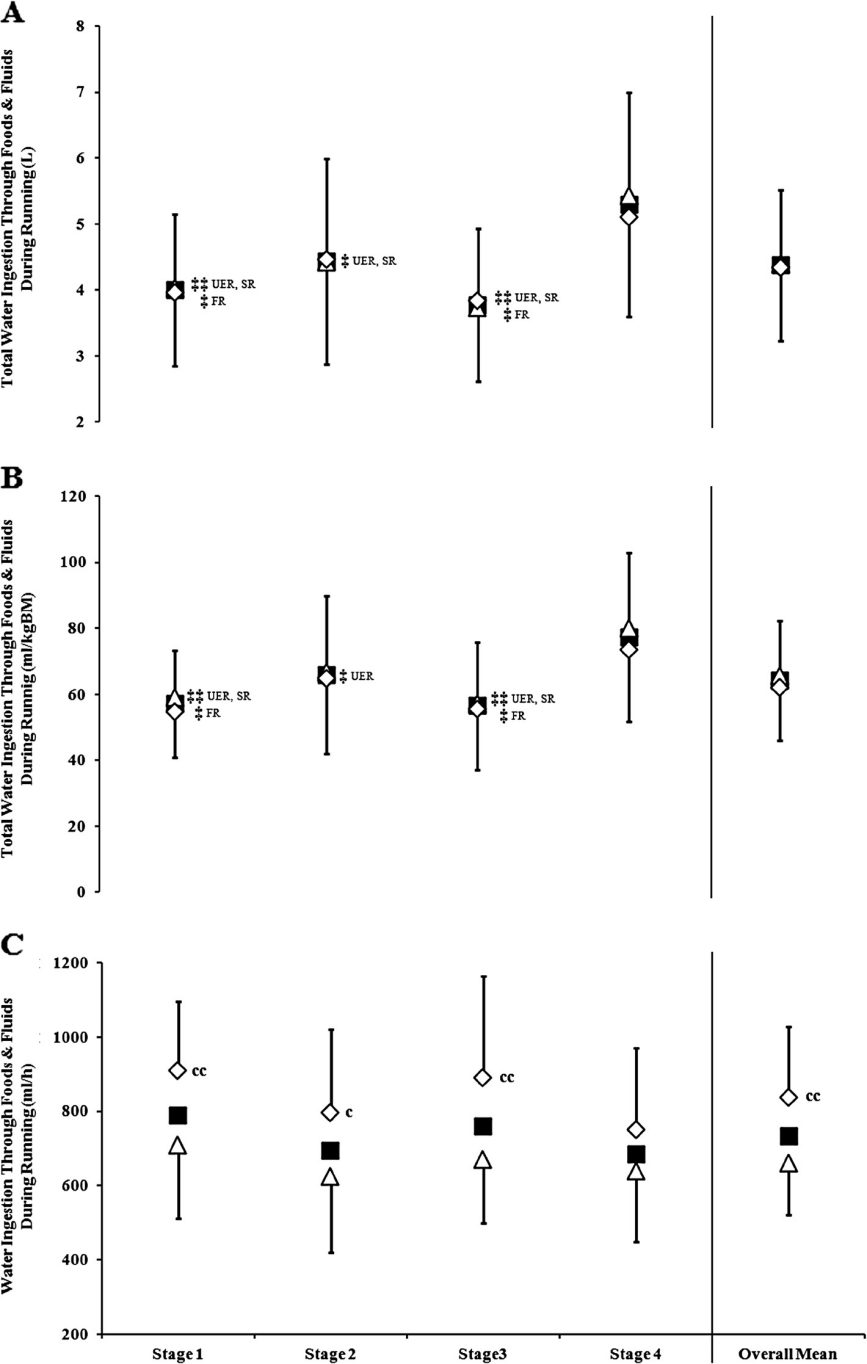
**Water ingestion habits during running of UER along MSUM competition.** (**A**) Total water ingestion, (**B**) Total water ingestion corrected for BM, (**C**) Rate of water ingestion. Mean (SD): UER (■, *n* = 54), slow runners (SR ∆; MSUM mean speed <8 km/h, *n* = 32), and fast runners (FR ◊; MSUM mean speed ≥8 km/h, *n =* 22). ‡‡ *p <* 0.01 and ‡ *p <* 0.05 *vs.* Stage 4; ^cc^*p <* 0.01 and ^c^*p* < 0.05 *vs.* SR.

Total water ingestion during running was lower on Stages 1, 2, and 3 in UER (*p* < 0.001) and SR (*p* < 0.001), and lower on Stages 1 and 3 in FR (*p* = 0.005), compared with Stage 4 (Figure 1A). Similar results were observed when corrected for BM (Figure 1B). No difference in water ingested (total and corrected for BM) during running was observed between SR and FR within all stages. Even though the rate of water ingestion during running did not differ between stages in UER, FR presented a higher water ingestion rate compared with SR in Stages 1 to 3 (*p* < 0.001; Figure 1C). Plain water accounted for 79% of total fluids consumed during running by UER (SR: 78%, FR: 80%); with remaining fluid ingestion coming from nutrient rich sources, which included (in order of highest consumed volume in UER): carbohydrate solutions, soft drinks, carbohydrate-protein solutions, and fruit juices. Additionally, 26% of UER reported consuming only plain water during running throughout the entire MSUM.

No difference was observed for post-stage water ingestion (total and corrected for BM) between stages for UER, SR and FR (Table 3). Whereas, total post-stage water ingestion was higher (*p* < 0.001) in FR compared with SR on Stages 1 and 3. However, when corrected for BM, no difference was observed between SR and FR within stages. Plain water accounted for 45% (SR 45%, FR 46%) of total fluids consumed post-stage by UER, with remaining fluid ingestion coming from nutrient rich sources, which included (in order of highest consumed volume in UER): soft drinks, fruit juices, carbohydrate solutions, carbohydrate-protein solutions, protein solutions, and milks.

When comparing male and female ultra-runners, no significant difference in total daily (male 7884 (1652) ml/day, female 7434 (1208) ml/day), pre-stage (male 899 (445) ml, female 784 (258) ml), during running (male 4429 (1313) ml, female 4289 (834) ml), and post-stage (male 1259 (569) ml, female 1145 (386) ml) water ingestion was observed between genders. When corrected for BM, female ultra-runners presented higher total daily (127 (20) ml/kgBM/day; *p* < 0.001) and during running (72 (14) ml/kgBM; *p* < 0.001) water ingestion on all stages compared with male ultra-runners (106 (27) ml/kgBM/day and 59 (19) ml/kgBM, respectively). However, rate of water ingestion during running was higher on all stage in male ultra-runners (777 (188) ml/h; *p* < 0.001) compared with female ultra-runners (661 (154) ml/h).

### Dietary sodium intake

Total daily sodium ingestion, through foods and fluids, did not vary between stages for UER (3.9 (1.3) g/day), SR (3.8 (1.3) g/day), and FR (4.2 (1.4) g/day) throughout the MSUM. Whereas, total daily sodium ingestion in CON was higher on Stage 4 (3.8 g/day) compared with Stages 1 to 3 (1.5 to 2.2 g/day; *p* < 0.000). Total daily sodium ingestion was lower in CON on Stages 1 to 3 compared with UER (*p* < 0.001), and was lower in SR on Stages 3 and 4 compared with FR (*p* = 0.048). When total daily sodium ingestion is represented as sodium intake per litre of fluid volume ingested, no significant difference was observed between stages for UER (564 (268) mg/L), SR (584 (304) mg/L), and FR (534 (207) mg/L), nor between SR and FR within stages; which were under benchmark recommendations. However, CON showed a higher sodium intake per litre of fluid volume ingested on Stage 4 (1133 mg/L) compared with Stages 1 to 3 (519 to 682 mg/L; *p* < 0.000), and compared with UER on Stage 4 only (549 mgNa/L; *p* = 0.007).

Total sodium ingestion and sodium intake per litre of fluid volume ingested pre-stage did not vary between stages for UER (947 (538) mg; 1209 (614) mg/L), SR (1040 (584) mg; 1281 (617) mg/L), and FR (811 (443) mg; 1104 (607) mg/L); nor between SR and FR within stages. Total sodium ingestion and sodium intake per litre of fluid volume ingested during running did not vary between stages for UER (1096 (630) mg; 271 (155) mg/L), SR (1092 (588) mg; 269 (133) mg/L), and FR (1102 (702) mg; 275 (184) mg/L), nor between SR and FR within stages; which were under benchmark recommendations. Total sodium ingestion and sodium intake per litre of fluid volume ingested post-stage did not vary between stages for UER (456 (313) mg; 440 (372) mg/L), SR (475 (316) mg; 498 (428) mg/L), and FR (428 (314) mg; 356 (259) mg/L), nor between SR and FR within stages; which were under benchmark recommendations. Moreover, overall daily, pre-stage, during running, and post-stage total sodium ingestion and sodium intake per litre of fluid volume ingested did not differ between genders in all stages, and were under benchmark recommendations.

### Serum sodium concentration

**Figure 2:**
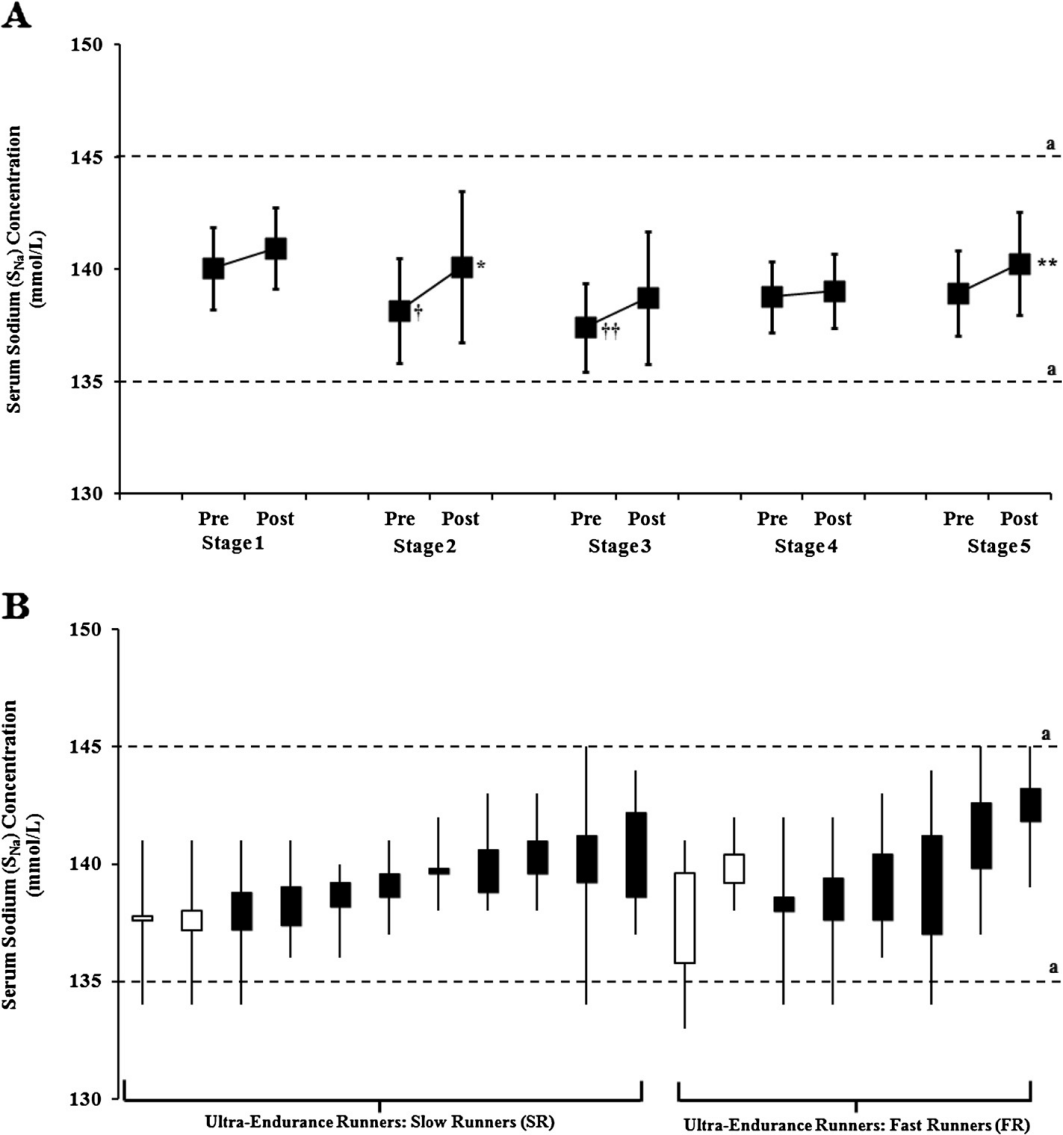
**Serum sodium (S**_**Na**_**) responses of UER along MSUM competition.** (**A**) S_Na_ concentration, (**B**) Individual S_Na_ responses. Mean (SD): UER (■, *n =* 19), slow runners (SR; MSUM mean speed <8 km/h, *n =* 11), and fast runners (FR; MSUM mean speed ≥8 km/h, *n =* 8). ^a^ Normal physiological range 135 to 145 mmol/L [32]; hyponatraemia <135 mmol/L [24]. Closed bars represent a pre- to post-stage increase in S_Na_ concentration. Open bars present a pre- to post-stage decrease in S_Na_ concentration. †† *p <* 0.01 and † *p <* 0.05 *vs.* pre-Stage 1; ^**^p < 0.01 and ^*^p < 0.05 *vs.* pre-stage.

Pre-stage S_Na_ concentration of sampled participants (*n* = 19) was lower on Stages 2 and 3, compared with Stage 1, in UER (*p* = 0.001; Figure 2A) and SR (*p* = 0.025), but did not change in FR. While, post-stage S_Na_ concentration of sampled participants did not differ in UER, SR, and FR between stages. Pre- and post-stage S_Na_ concentration did not differ between SR and FR within stages. Significant increases in pre- to post-stage S_Na_ concentration only occurred on Stages 2 and 5 in UER and SR (*p* = 0.001). S_Na_ concentration indicative of normonatraemia were evident in sampled UER along the course of the MSUM, with no episodes of hypernatraemia observed. However, S_Na_ concentrations indicative of hyponatraemia (<135 mmol/L) [24] were evident in *n* = 8 of UER (SR: *n* = 4, FR: *n* = 4) sampled along the MSUM (corresponding to 42% of the sampled population), occurring both pre- (Stages 1 to 4) and post-stage (Stages 2 to 4; Figure 2B). Moreover, pre- and post-stage S_Na_ concentration did not differ between male and female ultra-runners within stages along the MSUM. S_Na_ concentrations indicative of hyponatraemia was evident in both male and female ultra-runners (corresponding to 38% and 50% of sampled males and females, respectively) throughout the MSUM.

### Body mass

**Figure 3:**
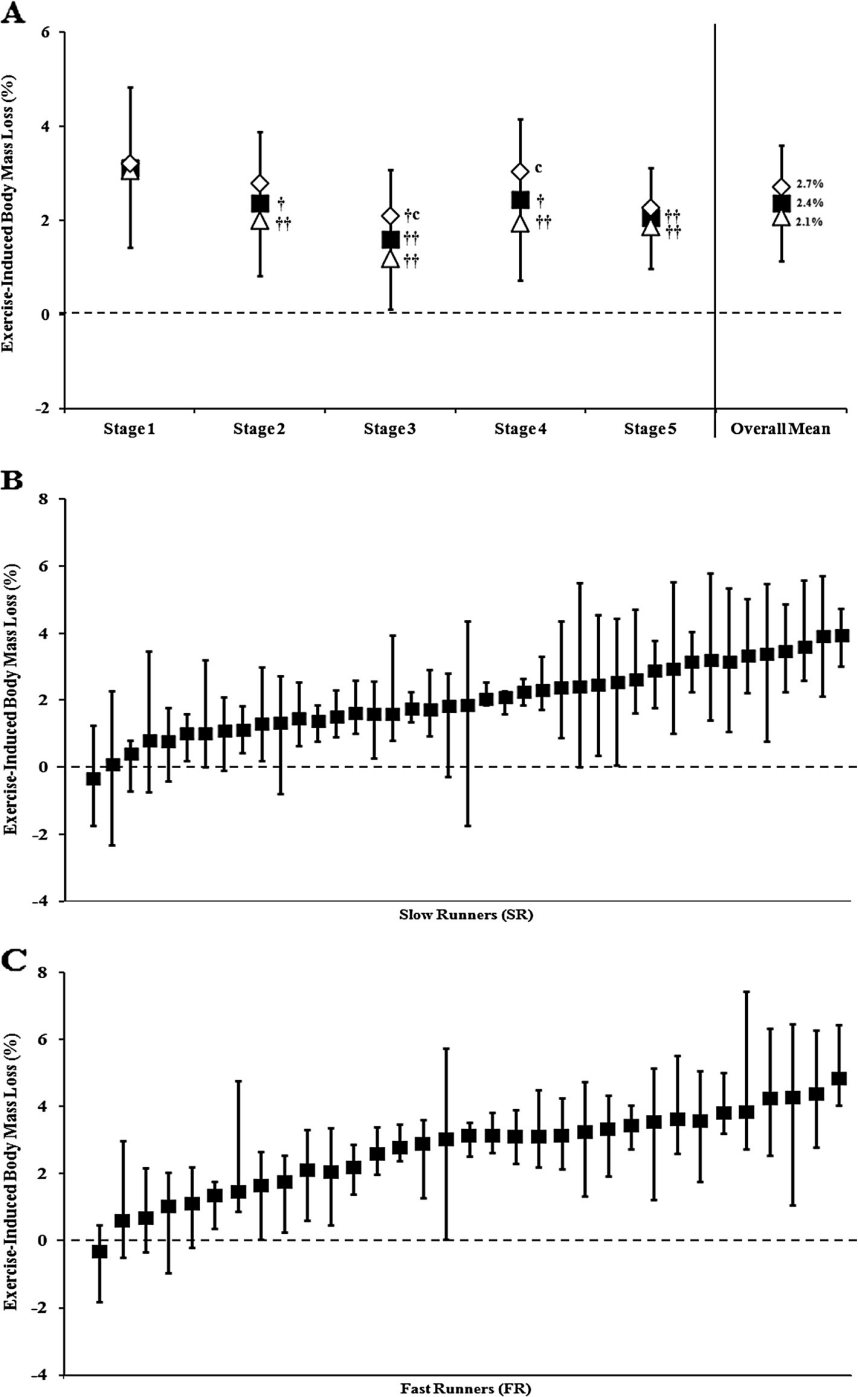
**Exercise-induced BM loss of UER along MSUM competition.** Mean (SD): (**A**) UER (■, *n =* 74); (**B**) slow runners (SR ∆; MSUM mean speed <8 km/h, *n =* 41); (**C**) fast runners (FR ◊; MSUM mean speed ≥8 km/h, *n =* 33). †† *p <* 0.01 and † *p <* 0.05 *vs.* Stage 1; ^c^*p <* 0.05 *vs.* SR.

Pre- and post-stage BM did not differ between stages in UER, CON (pre-stage only), SR, and FR throughout the MSUM. However, BM loss did occur from pre- to post-stage in UER, SR, and FR in all stages (pre-stage: UER 69.5 (11.0) kg, SR 67.8 (11.7) kg, FR 71.6 (9.9) kg; post-stage: UER 67.8 (10.6) kg, SR 66.3 (11.4) kg, FR 69.7 (9.4) kg). Stage 1 induced a greater BM loss compared with Stages 2 to 5 in UER (*p* < 0.001) and SR (*p* < 0.001), and Stage 3 only in FR (*p* = 0.021; Figures 3A). Additionally, FR showed a greater exercise-induced BM loss compared with SR in Stages 3 and 4 (*p* < 0.001). Pre- to post-stage BM gains along the course of the MSUM were evident in 29% of SR (Figure 3B), and 21% of FR (Figure 3C). Moreover, exercise-induced BM loss was greater in male ultra-runners (*p* < 0.001) on Stages 1 to 3 (3.4 (1.7)%, 2.8 (1.4)%, and 1.9 (1.4)%, respectively) compared with female ultra-runners (2.6 (1.7)%, 1.5 (1.6)%, and 1.0 (1.5)%, respectively). Pre- to post-stage BM gains was evident in both genders (males 22%, females 32%).

### Plasma osmolality, plasma volume change, and body water

Pre- and post-stage P_Osmol_ did not differ between stages in UER, CON (pre-stage only), SR, and FR; and between UER and CON, nor SR and FR within stages throughout the MSUM (Table 4). P_Osmol_ increased pre- to post-stage in UER (*p* < 0.001), SR (*p* < 0.001), and FR (*p* < 0.001) on all stages. In UER, mean P_Osmol_ remained within normal clinical reference range (280–303 mOsmol/kg) [32] throughout the MSUM, except on pre-Stages 3 and 4 (under normal clinical reference range). P_Osmol_ was higher in male ultra-runners on pre-Stage 2 only (288 (14) mOsmol/kg; *p* = 0.031) compared with female ultra-runners (274 (18) mOsmol/kg). No significant difference in post-stage P_Osmol_ was observed between genders.

**Table 4:** **Plasma (P**_**Osmol**_**) and urine (U**_**Osmol**_**) osmolality, and U**_**Osmol**_**/P**_**Osmol**_**ratio of UER along MSUM competition**

	**Stage 1**		**Stage 2**		**Stage 3**		**Stage 4**		**Stage 5**	
	**Pre**	**Post**	**Pre**	**Post**	**Pre**	**Post**	**Pre**	**Post**	**Pre**	**Post**
Plasma Osmolality (mOsmol/kg)										
UER	281 (16)	287 (14)^*^	283 (16)	297 (17)^*^	272 (13)	291 (16)^**^	271 (12)	298 (10)^**^	283 (15)	296 (10)^**^
CON	287 (13)				276 (14)				270 (9)	
SR	280 (18)	286 (17)^*^	282 (17)	294 (16)^*^	273 (15)	287 (16)^**^	270 (10)	298 (6)^**^	286 (13)	294 (10)^*^
FR	282 (12)	289 (9)^*^	285 (11)	302 (18)^**^	271 (9)	297 (14)^**^	273 (14)	299 (14)^**^	278 (17)	298 (11)^**^
Urine Osmolality (mOsmol/kg)										
UER	535 (278)	811 (261)^**^	673 (311)	914 (367)^**^	792 (306)^††b^	997 (352)^**†^	750 (293)^††^	898 (333)^**^	834 (343)^††^	1050 (402)^**††^
CON	577 (197)				545 (78)				608 (349)	
SR	514 (295)	840 (226)^**^	702 (326)	969 (393)^**^	849 (294)^††^	1075 (356)^**††^	770 (301)^††^	939 (323)^**^	846 (355)^††^	1086 (400)^**††^
FR	562 (256)	775 (298)^**^	636 (293)	844 (324)^**^	722 (311)	899 (326)^**c^	724 (285)	847 (344)^**^	818 (334)^†^	1004 (406)^**^
Urine to Plasma Osmolality ratio										
UER	2.0 (0.6)	2.7 (0.9)^**^	2.6 (1.0)	3.3 (1.1)^**^	3.3 (1.0)^††bb^	3.9 (1.2)^††**^	3.1 (1.0)^††^	3.3 (1.0)^**^	3.5 (1.1)^††bb^	4.2 (1.3)^††**^
CON	2.0 (0.3)				2.0 (1.0)				2.2 (1.2)	
SR	2.0 (0.7)	2.8 (0.9)^**^	2.8 (1.0)	3.7 (1.3)^**cc^	3.5 (0.9)^††^	4.2 (1.1)^††**^	3.2 (1.0)^††^	3.3 (1.0)^**^	3.6 (1.1)^††^	4.4 (1.2)^††**^
FR	2.1 (0.6)	2.6 (0.9)^**^	2.1 (1.0)	2.8 (1.3)^**^	3.1 (1.0)^†^	3.5 (1.1)^**^	3.1 (1.0)^†^	3.3 (0.9)^**^	3.4 (1.3)^††^	4.0 (1.5)^††**^
Individual UER response range (U_Osmol_/P_Osmol_ ratio)										
	0.6 to 2.9	0.9 to 4.0	0.4 to 4.6	0.5 to 6.1	1.3 to 4.8	1.6 to 6.1	1.1 to 4.7	1.1 to 5.0	0.9 to 5.4	1.1 to 6.6

Mean (SD): UER (U_Osmo_*n =* 72, P_Osmol_*n* = 28); Control (CON, *n =* 12); slow runners (SR; MSUM mean speed <8 km/h, U_Osmo_*n =* 40, P_Osmol_*n* = 17); fast runners (FR; MSUM mean speed ≥8 km/h, U_Osmo_*n =* 32, P_Osmol_*n* = 11). †† *p <* 0.01 and † *p <* 0.01 *vs.* Stage 1; ^**^p < 0.01 and ^*^p < 0.05 *vs.* pre-stage; ^bb^*p <* 0.01 and ^b^*p <* 0.05 *vs.* CON; ^cc^*p <* 0.01 and ^c^*p <* 0.05 *vs.* SR.

From baseline (pre-Stage 1), a significant increase in P_V_ was evident pre-Stage 3 onwards in UER (+9.7%), SR (+8.0%), and FR (+12.0%; *p* < 0.001); peaking at pre-Stage 5 (+20.4%, +18.6%, and +22.8%, respectively). No significant change in P_V_ was observed in CON. UER presented a significantly higher P_V_ than CON (*p* = 0.001) pre-Stages 3 (+6.2%) and 5 (+18.6%). No difference in P_V_ was observed between SR and FR, nor between genders, within stages throughout the MSUM.

**Figure 4:**
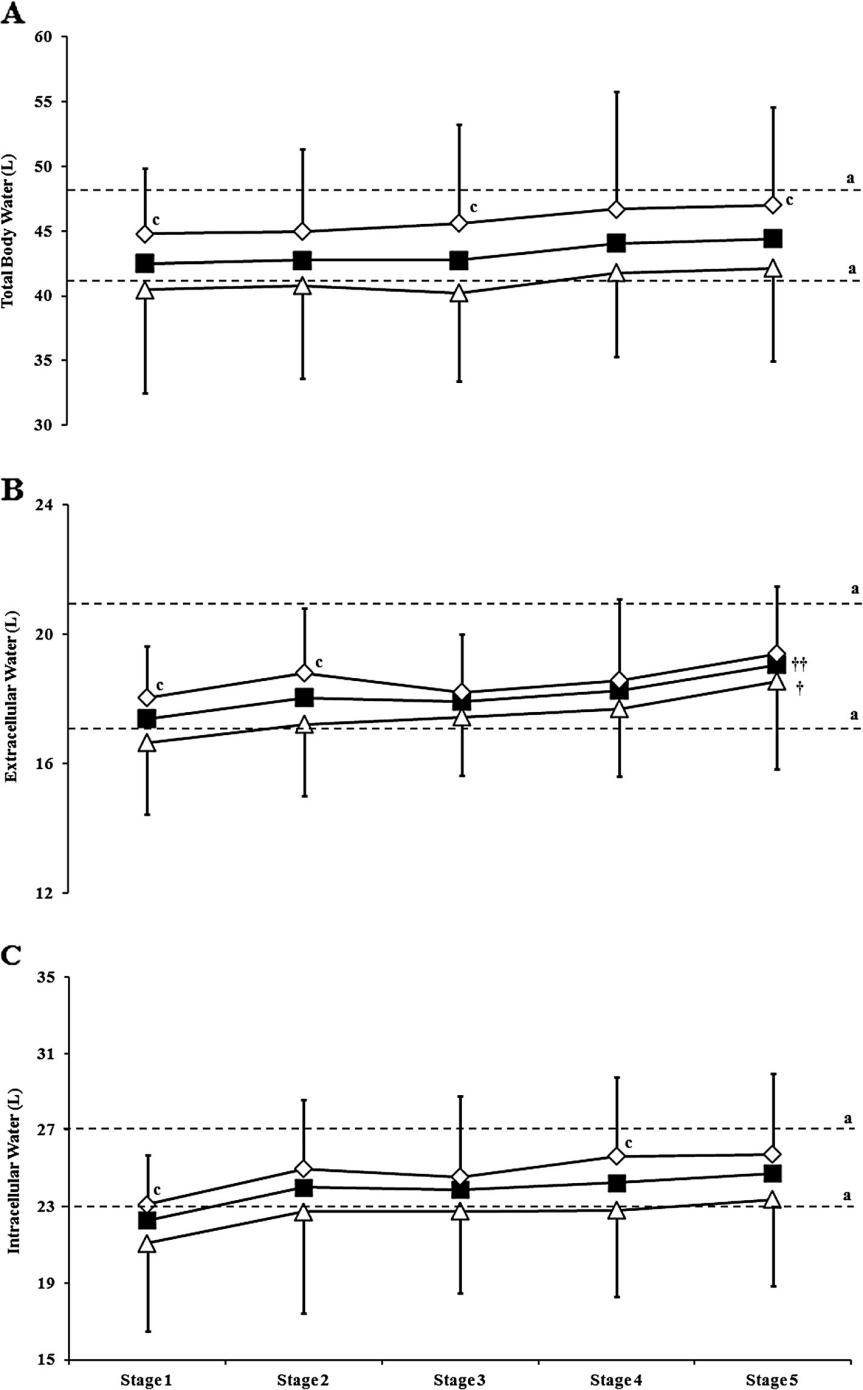
**Changes in pre-stage resting body water of UER along MSUM competition.** (**A**) Total body water, (**B**) Extra-cellular water, (**C**) Intra-cellular water. Mean (SD): UER (■, *n =* 43); slow runners (SR ∆; MSUM mean speed <8 km/h, *n =* 23); fast runners (FR ◊; MSUM mean speed ≥8 km/h, *n =* 20). ^a^ Normal physiological range (Quadscan 4000, Bodystat, Douglas, Isle of Man, UK). †† *p <* 0.01 and † *p <* 0.05 *vs.* Stage 1; ^c^*p <* 0.05 *vs.* SR.

Pre-stage total body water did not differ between stages in UER, SR, and FR (Figure 4A). SR presented a lower pre-stage total body water on Stages 1, 3 and 5 (*p* < 0.001) compared with FR. Pre-stage extra-cellular water was significantly higher on Stage 5 compared with Stage 1 in UER (*p* = 0.012) and SR (*p* = 0.05), but did not differ between stages in FR (Figure 4B). SR presented lower pre-stage extra-cellular water on Stages 1 and 2 (*p* < 0.001) compared with FR. Pre-stage intra-cellular water did not differ significantly between stages in UER, SR, and FR (Figure 4C). SR presented lower pre-stage intra-cellular water on Stages 1 and 4 (*p* < 0.001) compared with FR. Pre-stage total body, extra-cellular, and intra-cellular water was higher (*p* < 0.001) in male ultra-runners on all stage (46.8 (5.9) L, 19.1 (1.8) L, and 26.1 (2.8) L, respectively) compared with female ultra-runners (36.7 (5.2) L, 16.2 91.8) L, and 19.4 (3.4) L, respectively). However, when corrected for BM, no significant difference in pre-stage total body, extra-cellular, and intra-cellular water was observed between SR and FR, nor between male and female ultra-runners.

### Urine measures

Pre-stage U_Colour_ was darker on Stages 2 to 5 in UER (4 to 6), SR (4 to 6), and FR (4 to 6) compared with Stage 1 (3 (1)); but did not significantly change in CON (3 (1)) along the MSUM. UER presented a darker U_Colour_ on pre-Stages 3 and 5 compared with CON (*p* = 0.004); whereas, no difference in pre-stage U_Colour_ was observed between SR and FR within stages. No difference in post-stage U_Colour_ was observed between stages in UER (6 (1)), SR (6 (1)), and FR (6 (1)); nor between SR and FR within stages. Increases darkness in U_Colour_ (*p* < 0.001) were observed pre- to post-stage on all stages for UER, SR, and FR. Moreover, no significant difference in pre- and post-stage U_Colour_ was observed between male and female ultra-runners.

Pre-stage U_Osmol_ was higher on Stages 3 to 5 in UER (FR (*p* < 0.001) and SR (*p* < 0.001), and Stage 5 only in FR (*p* = 0.01), compared with Stage 1; but did not significantly change in CON (Table 4). UER presented a higher U_Osmol_ on pre-Stage 3 only compared with CON (*p* = 0.008). Post-stage U_Osmol_ was higher on Stages 3 and 5 in UER (*p* < 0.001) and SR (*p* = 0.009), compared with Stage 1; but did not differ between stages in FR. Increases in U_Osmol_ were observed pre- to post-stage on all stages for UER (*p* < 0.001), SR (*p* < 0.001), and FR (*p* < 0.001). Moreover, a higher pre- (*p* < 0.001) and post-stage (*p* = 0.008) U_Osmol_ was observed on Stage 5 only in males ultra-runners (907 (326) mOsmol/kg, 1126 (392) mOsmol/kg, respectively) compared with female ultra-runners (697 (339) mOsmol/kg, 905 (387) mOsmol/kg, respectively).

Similarly, pre-stage U_Osmol_/P_Osmol_ ratio was higher on Stages 3 to 5 in UER (*p* < 0.001), SR (*p* < 0.001), and FR (*p* = 0.022), compared with Stage 1; but did not significantly change in CON (Table 4). UER presented a higher U_Osmol_/P_Osmol_ ratio on pre-Stages 3 and 5 compared with CON (*p* < 0.001); whereas did not differ between SR and FR within stages. Post-stage U_Osmol_/P_Osmol_ ratio was higher on Stages 3 and 5 in UER (*p* < 0.001) and SR (*p* < 0.001), and Stage 5 only in FR (*p* = 0.049), compared with Stage 1. A higher U_Osmol_/P_Osmol_ ratio was observed in SR compared with FR on post-Stage 2 only (*p* = 0.046). Increases in U_Osmol_/P_Osmol_ ratio were observed pre- to post-stage on all stages for UER (*p* < 0.001), SR (*p* < 0.001), and FR (*p* = 0.038). Moreover, pre-stage U_Osmol_/P_Osmol_ ratio was higher in male ultra-runners on Stages 3 to 5 (3.7 (0.8), 3.4 (0.9), and 3.6 (0.9), respectively; *p* < 0.001) compared with female ultra-runners (2.5 (1.0), 2.4 (0.8), and 2.5 (1.2), respectively). While, post-stage U_Osmol_/P_Osmol_ ratio was also higher in male ultra-runners on Stages 3 to 5 (4.3 (1.0), 3.6 (0.9), and 4.6 (1.1), respectively; *p* < 0.001) compared with female ultra-runners (3.0 (1.1), 2.6 (0.8), and 3.1 (1.3), respectively).

## Discussion

The aims of the current study were, firstly to assess water and sodium intake habits of recreational ultra-runners during a semi self-sufficient MSUM conducted in a hot ambient environment; secondly to monitor serum sodium concentration, and hydration status using multiple hydration assessment techniques, along competition. In contrast to our hypothesis, water intake habits in the majority of UER were sufficient to maintain baseline euhydration levels whilst competing on consecutive days in hot ambient conditions. Despite sodium ingestion through foods and fluids under benchmark recommendations in the majority of UER, in accordance with our hypothesis, normonatraemia was observed in all UER along the MSUM. However, a novel finding was evidence of fluid over-consumption behaviours in a substantial number of UER, irrespective of running speed or gender, with asymptomatic hyponatraemia observed at certain points along the MSUM in *n* = 8 UER (corresponding to 42% population). The strength of the sample size (equating to 55% of all runners that participated in the MSUM), potentially gives a valid and reliable representation of current water and sodium intake habits and status of ultra-runners during semi self-sufficient MSUM conducted in hot ambient conditions, bearing in mind the diverse origins of the sampled population. Conversely, the extreme nature of the event and invasive techniques used to determine blood borne variables at frequent points along the MSUM created a potential barrier for obtaining full data sets from all participants. In light of this, only a sub-sample of the full population was used in determining blood borne variables.

Average total daily water ingestion through foods and fluids of UER was 7.7 L/day, and appears to be sufficient to maintain baseline euhydration levels throughout the current semi self-sufficient MSUM, in accordance with the maintained P_Osmol_ within normal clinical reference range both pre- and post-stage in the majority of UER. This *ad libitum* rate of water ingestion also provided a surplus of water required to supported the acute extracellular hypervolaemia observed in both SR and FR along competition (as indicated by 22.8% P_V_ and 9.2% ECW increase in UER). This response is likely attributed to heat acclimatisation [5], since 72% of UER originated from countries that presented cold or thermoneutral environmental conditions (≤20°C), and arrived at the competition location <48 h prior the start of competition, without having previously heat acclimatised. These results also highlight practical relevance for self-sufficient MSUM, which normally provide water rations of ~12 L/day; suggesting that even with a water quota set by race organisers, euhydration can easily be maintained throughout competition. However, the quality of fluids ingested by UER could be adjusted, since the majority of fluid consumed was plain water, with nutrient rich fluids only accounting for ≤30% of overall fluid intake along the course of the MSUM. Educating UER to predominantly consume nutrient rich fluids throughout competition will contribute towards both euhydration and cater for meeting energy and nutritional needs on consecutive days of strenuous exercise [39].

Average total water intake through foods and fluids during running in UER was 4.5 L, with FR and male ultra-runners showing ability to tolerate higher rates of fluid consumption than SR and female ultra-runners, respectively. Thirst and appetite sensations, gastrointestinal distress during running, concomitant with training status and degree of exposure to competition beverages during training, are all are prime factors to fluid tolerance during exercise-heat stress [8,11,40,41]. The majority of fluid consumed during running was plain water, with nutrient rich fluids only accounting for ≤26% of overall fluid intake. Such drinking behaviours during running are prime risk factor for hyponatraemia [17,24,26], with prevalence on hyponatraemia observed during a one stage 161 km running event [42]; and now during a 5 stage 225 km ultra-marathon in the heat, with 26% of UER sampled presenting exercise-associated hyponatraemia post-stage along the MSUM, irrespective of running speed, running distance, and/or gender; with incidence not accumulating over the MSUM. The interesting feature of the current study was that S_Na_ indicative of hyponatraemia also occurred pre-stage (16% of UER sampled). Therefore, over-consumption of plain water during the recovery period and/or sub-optimal dietary sodium intakes may have also attributed to the incidences of asymptomatic hyponatraemia observed [6,18,19,26]. Indeed, even though the majority of UER were actively adding external sodium supplementation to ingested fluids, sodium intake per ingested fluid volume during running and the recovery period were far below benchmark recommendation throughout the entire MSUM in UER [6,18]. Conversely, despite the low sodium concentrations per fluid volume ingested, 58% of UER sampled remained normonatramic throughout the entire MSUM. These results are in accordance with previous studies suggesting that sodium supplementation may not be required during exercise in certain UER, since adaptations to increase sodium bioavailability and prevent losses (e.g. sweat, urine, and faeces) take place in response to periods of sodium deprivation or restriction [14,23,24].

BM loss ≥2% has previously been established as indicative of dehydration [1]. In the current study the average exercise-induced BM loss in UER was 2.4%, which is relatively low compared with reports of >4% BM loss often observed after marathon and one-stage ultra-marathon competition [17]. Moreover, FR and male ultra-runners showed a greater exercise-induced BM loss compared with SR and female ultra-runners, respectively. This is in accordance with previous reports indicating highly trained faster runners lose more BM during competition than lesser trained slower runners [16,17]; but also present novel finding, showing that male ultra-runners lose more BM than female ultra-runner, possibly associated with running speed. Nevertheless, these results support the suggestion that SR, and now female ultra-runners, have the increased tendency for fluid over-consumption; with BM gains observed pre- to post-stage in 29% and 32% of SR and female ultra-runners respectively, compared with 21% and 22% of FR and male ultra-runners respectively. BM loss results from a combination of factors during the run phase of MSUM competition in the heat, including: water sweat, respiratory, and urine losses (taking into account potential gastrointestinal fluid reservoirs, awaiting intestinal transport, during the pre-stage period), weight loss associated with depletion of muscle glycogen stores, and/or faecal weight loss if evacuation is required during running [2,6,29]. Therefore, care is needed in using the degree of exercise-induced BM loss to programme fluid intake strategies during post-stage recovery, to correctly replace water sweat and obligatory urine losses. Water replacement rates after exercise of x1.5 BM loss have been recommended, and appear to be an effective and safe in replenishing exercise-heat stress induced body water losses [1]. Using nutrient rich fluids during the post-stage recovery period (e.g. milk shake), will provide a plethora of essential ingredients required for an overall effective recovery [43-47].

The novel use of MBIA in the current field setting showed that euhydration was maintained throughout the entire course of the MSUM, with pre-stage hydration status actually improving along competition. The observed 9.2% increase in resting pre-stage ECW from pre-Stage 1 to pre-Stage 5 in UER, are likely associated with heat acclimatisation responses promoting acute extracellular hypervolaemia [48-50]. Exercise-heat stress raises circulatory osmotic pressure stimulating a vascular influx of plasma proteins (e.g. albumin) which draws fluid into intravascular compartments, and up-regulates hormones (e.g. aldosterone, vasopressin) responsible for renal water re-absorption [5,51]. Even though P_V_ expansion was not directly measured, using change in blood haemoglobin and haematocrit has previously been used as a valid method of estimating changes in P_V_ during exercise-heat stress [16,35-37]. Increases in P_V_ were observed in the current MSUM in UER, but not in CON, which are in accordance with ultra-endurance specific heat acclimation protocols, reporting significant increases in P_V_ (+7.9%) after two bouts of 2 hours running at 60% VO_2max_ at 30°C [35]. These results imply that simply starting MSUM competition in the heat will support a positive effect on hydration status if sufficient fluids are consumed to accompany heat adaptation responses.

Using urine measure of hydration to guide fluid intake and support euhydration is a common practice amongst ultra-endurance athletes during multi-stage competitions. Whereby, ultra-endurance athletes consume fluids based on their urine concentration (e.g. colour, osmolality). In the current study, pre-stage U_Osmol_ and U_colour_ rose substantially by Stage 2 and remained elevated throughout the MSUM in UER, with no change on CON; whilst the high U_Osmol_ values post-stage are a common feature of exercise-heat stress [28]. However, the substantial increase in U_Osmol_/P_Osmol_ ratio above clinical reference values in the majority of UER, irrespective of running speed and gender, at both pre- and post-stage, concomitant with progressive increases in P_V_ and ECW observed as competition progressed, indicates U_Osmol_ does not reflect body water content; suggesting urine measure of hydration are potentially inappropriate in monitoring hydration status and guiding fluid intake strategies in UER during MSUM in the heat. Moreover, all UER who presented asymptomatic hyponatraemia and BM gains post-stage had U_Osmol_/P_Osmol_ ratios above clinical reference values. Interestingly, male ultra-runners presented greater U_Osmol_/P_Osmol_ ratios compared with female ultra-runners Stage 3 onwards, likely due to the greater fluid over-consumption behaviours of female ultra-runners. However, differences in renal hormone responses between genders to exercise-heat stress should not be overlooked, and warrants further investigation. Even though a limitation in the current study is the absences of data on renal hormone responses during MSUM conducted in a hot ambient environment, previous ultra-endurance studies have reported a four-fold increase in vasopressin during a 109 km cycle race in the heat [25]. Thus, from a practical standpoint, using results from urine measure of hydration techniques to guide hydration strategies during periods of exercise-heat stress could actually be dangerous for certain individuals, promoting fluid over-consumption (attempts to maintain clear urine) while renal water re-absorption is up-regulated [22]. Using thirst as a mechanism to regulate fluid intake behaviour both during running and recovery appears to be a reliable and safer method in promoting euhydration maintenance along MSUM competition in the heat [13-15].

## Conclusion

Water intake habits of recreational ultra-runners appear sufficient to maintain baseline euhydration levels during consecutive days of prolonged strenuous exercise in hot ambient conditions. The observed high intakes of plain water, pre- to post-stage BM gains, evidence of asymptomatic hyponatraemia both pre- and post-stage, concomitant with the progressive increases in pre-stage body water as competition progressed suggests prevalence of fluid over-consumption behaviours in a substantial number of recreational ultra-runners, irrespective of running speed, running distance, and/or gender. Normonatraemia was observed along the MSUM in the majority of UER, despite sodium ingestion under benchmark recommendations. These findings suggest that relevant hydration education, which may include: adherence to a heat acclimation protocol in the week leading up to MSUM, ‘*drink to thirst*’ , selection and ingestion of nutrient rich fluids during running and recovery, and increase the consumption of sodium rich foods during the recovery period, may contribute towards encouraging ultra-runners to establish more appropriate hydration optimising behaviours during future MSUM events. These improved behaviours should not only support euhydration and endurance running performance, but also mitigate the development of clinically significant episodes arising from both dehydration and over-hydration.

## Abbreviations

ASL: Above sea level; BM: Body mass; CON: Control; FR: Fast runners; MSUM: Multi-stage ultra-marathon; P_Osmol_: Plasma osmolality; P_v_: Plasma volume; RH_max_: Maximum relative humidity; SR: Slow runnrs; T_max_: Maximal daily ambient temperature; UER: Ultra-endurance runners; U_Colour_: Urine colour; U_Osmol_: Urine osmolality.

## Competing interests

The authors declare that they have no competing interests.

## Authors’ contributions

RC was responsible for the original research idea, overall supervision and management of the research project. RC, AT, LR, VS, AM, and CT contributed towards the development of the experimental design. RC, AT, LR, AS, LH, BL, VC, SG, JW, EF, ED, JH, SM, EV, VS, and AM contributed towards various aspects of data collection and sample analysis. RC and SG contributed towards analysis of the raw data. RC and CT contribute towards the preparation and review of the manuscript. All authors read and approved the final manuscript.
